# Thermo-Hydric Study of Wood-Based Materials under Thermal Comfort Conditions

**DOI:** 10.3390/ma17051177

**Published:** 2024-03-02

**Authors:** Mohamed Haddouche, Fahed Martini, Mounir Chaouch, Adrian Ilinca

**Affiliations:** 1 Wind Energy Research Laboratory (WERL), Université du Québec à Rimouski, UQAR, Rimouski, QC G5L 3A1, Canada; mohamed.haddouche@uqar.ca; 2 Mechanical Engineering Department, École de Technologie Supérieure, Montreal, QC H3C 1K3, Canada; fahed.martini@etsmtl.ca; 3 Service de Recherche Et D’Expertise En Transformation Des Produits Forestiers, SEREX, Amqui, QC G5J 1K3, Canada; mounir.chaouch@serex.ca

**Keywords:** moisture content, thermal comfort, air relative humidity, drying time

## Abstract

This paper tackles the issue of moisture variation in wood-based materials, explicitly focusing on melamine-coated particleboard (hereafter referred to as melamine) and medium-density fiberboard (MDF) used in the third phase of wood industry transformation. The approach involves a comprehensive strategy for predicting moisture content variation, incorporating numerical simulation, experimental testing, and the application of artificial neural network (ANN) technology to enhance accuracy in furniture manufacturing. The developed ANN models are tailored to predict moisture content changes under specific thermal comfort conditions. Remarkably, these models demonstrate high precision, with an average error margin of only 1.40% for 8% moisture content (MC) and 2.85% for 12% MC in melamine, as well as 1.42% for 8% MC and 2.25% for 12% MC in MDF. These levels of precision surpass traditional models, emphasizing this study’s novelty and practical relevance to the industrial context. The findings indicate that ANN models adapt to diverse environmental conditions, presenting a robust tool for optimizing moisture management in wood-based materials. This research contributes valuable insights for improving the reliability and efficiency of moisture content predictions in the wood industry.

## 1. Introduction

Wood, a versatile natural resource, plays a dual role as both an energy generator when used as biomass and an energy consumer during processing activities, such as the machining of wood-based materials [1]. Drying, a crucial step in wood transformation, constitutes a significant portion (40% to 70%) of the total energy used in wood product manufacturing [2]. The energy consumption in the drying process stems from heating and air/product transportation through the dryer. Therefore, adequate heating, air circulation, and product throughput management are vital for enhancing energy efficiency [3]. Optimizing these energy-intensive processes, especially drying, is fundamental to reducing energy consumption [4]. However, improper storage of wood-based materials increases their moisture content (MC), leading to higher energy consumption, costs, and greenhouse gas emissions associated with drying [5,6].

The main strategies employed to reduce energy consumption in wood product drying include [3]:

- Developing new general methods;
- Utilizing alternative energy sources and minimizing heat losses to the atmosphere;
- Implementing low-cost methods, such as artificial intelligence or simulations, for potential energy savings;
- Coordinating processes for efficient energy use;
- Emphasizing environmentally friendly solutions and reducing greenhouse gas emissions.

While previous studies often focus on reducing energy consumption during high-temperature drying, especially for lumber [2,6], or address moisture transfer in wood-based materials during manufacturing, this research focuses on the ambient temperature behavior of wood-based materials. This distinctive approach underscores the novelty of our study, aiming to bridge the theoretical and practical knowledge gap in moisture management at ambient conditions.

This study investigates moisture transfer in two wood-based materials, MDF and melamine, under ambient temperature and air humidity conditions. This study consolidates empirical and modeled findings through experimental investigations and the subsequent development and validation of an artificial neural network (ANN) model, followed by numerical simulations.

For this study, “melamine” refers to melamine-faced boards or panels. These engineered wood products consist of a core substrate, such as particleboard or MDF (medium-density fiberboard), coated with a layer of melamine resin for surface finishing. Melamine resin, a durable thermosetting polymer synthesized from the melamine chemical compound, imparts a durable, moisture-resistant, and aesthetically appealing surface to these boards. The distinction between the raw melamine compound and its application in the form of melamine resin on wood-based substrates is critical, as this research focuses on the latter’s implications for moisture management and energy efficiency in wood product manufacturing.

Wood-based materials, such as MDF and melamine, are extensively used in the furniture industry. These materials offer advantages such as dimensional stability, machining capability, cost-effectiveness, and aesthetic versatility. The production capacity using these materials continues to increase annually [7]. However, concerns arise regarding the storage of raw materials (panels), particularly when shared storage areas with workshops must adhere to specific temperature and air humidity conditions to ensure user comfort, commonly known as “thermal comfort”, as mandated by health and safety regulations. On the flip side, the moisture content (MC in %) of wood panels ideally should range between 8% and 12% for proper machining, a criterion that is not always met. Consequently, wood-based materials necessitate drying under thermal comfort conditions. Natural drying, a simple and economical technique, reduces MC, minimizes installation costs, and utilizes minimal energy resources [8]. Therefore, this research is motivated by the need to ensure optimal moisture content (MC) for machining, which is often compromised due to suboptimal storage conditions. It employs a multifaceted approach to study the thermo-hydric behavior of wood-based materials and make comparisons. The methodology is as follows:

- Theoretical foundations and mathematical modelling: Describing phenomena within materials by extrapolating prior research to our context. A new formulation is developed to describe moisture transfer phenomena (diffusivity) in wood-based materials under specific thermal comfort conditions, subsequently used in the simulation.
- Numerical simulation and analysis: Visualize these phenomena and determine the required drying time to reach specific MC values (12% and 8%). Wood-based materials are assumed to be porous media, and diffusivity is derived from the first approach.
- Experimental study: Tracking MC evolution in two types of wood-based materials (melamine and MDF samples) over drying time for each extreme condition of the thermal comfort zone. The drying time for specific MC values is deduced.
- Artificial neural network (ANN) implementation: Combined with experimental data to build a generalized model calculating drying time under any environmental condition in the thermal comfort zone. The numerical model in MATLAB calculates the required time to reach 12% and 8% MC values. Drying time results are compared by calculating the percent error (PE) and subsequently discussed.

This study yields significant findings, advancing the understanding of moisture behavior in wood-based materials under thermal comfort conditions. The newly developed ANN model effectively and accurately predicts drying times, offering an efficient approach to managing moisture content in melamine and MDF. Furthermore, this research bridges the gap between theoretical models and practical applications, providing crucial insights into the natural drying process under varying environmental conditions. These contributions optimize manufacturing processes in the furniture industry and have broader implications for sustainable practices and energy conservation in wood product manufacturing.

The framework of the implemented multifaceted approach comprises four phases: the development of a theoretical mathematical model, the execution of numerical simulations, comprehensive empirical investigations, and the innovative application of artificial neural network (ANN) models. These methodologies collectively facilitate a comprehensive analysis of moisture behavior in wood-based materials, focusing on melamine and MDF and illuminating their behavior under different furniture manufacturing thermal comfort conditions.

In summary, this paper explores the complex landscape of moisture fluctuation in wood-based materials, highlighting melamine and MDF. By combining theoretical foundations, experimental investigation, and advanced numerical simulation culminating with cutting-edge ANN model implementation, this research provides new insights into moisture management optimization, particularly regarding practical applications within the specified thermal comfort parameters.

## 2. Detailed Problem Definition and Research Justification

After introducing the significance of optimizing energy consumption and improving moisture management in wood product manufacturing and the novelty of focusing on ambient temperature behavior, this section delves into the specific challenges associated with moisture variation in melamine and MDF. This exploration sets the groundwork for our investigative approach, directly addressing the research needs and objectives previously outlined.

### 2.1. Phenomenon Description

Like many other natural materials, wood and its derivatives exhibit hygroscopic properties, enabling them to absorb and release moisture from their environment. The moisture exchange between wood and air depends on the relative humidity (RH), the air temperature (T), and the current amount of water in the wood, which is referred to as moisture content (MC) [9,10].

**Figure 1:**
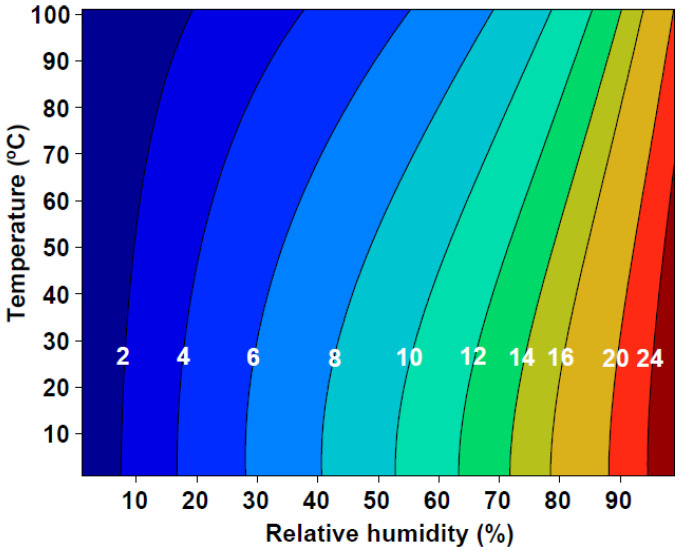
Example of equilibrium moisture content (%) for wood as a function of temperature (°C) and relative humidity (%) [9].

For wood composite materials, the relationships between equilibrium moisture content (EMC), relative humidity (RH), and temperature have great importance [11,12] because they affect the mechanical properties of materials. These properties include strength, dimensional stability, machinability, adhesiveness, and decay resistance [12,13]. This equilibrium between EMC, RH, and temperature can be illustrated as sorption isotherms (Figure 1) [12]. Therefore, moisture sorption (adsorption and desorption) is critical data for wood-based panels [10]. Q. Wu [12] demonstrates that the lower adsorption curve, compared to the desorption curve, indicates a reduced MC value at a constant RH level when approached from adsorption.

Therefore, controlling the moisture content before transformative processes, such as cutting, machining, and drilling, is crucial for ensuring high-quality outcomes. This includes dimensional stability and surface roughness while preventing deformation, swelling, and shrinking. Therefore, the moisture content of wood-based materials is recommended to fall within the range of 8% to 12% [14]. To achieve these optimal values, the panels are stored in ambient air conditions at 21 °C (70 °F) with a relative humidity ranging between 35% and 45% for 7 to 15 days. Additionally, achieving a lower equilibrium moisture content (EMC) in wood-based materials prevents minor dimensional alterations and the onset of biotic agents [15].

The drying process of furnishings (particles/fibers) differs significantly from solid wood [16]. The complexity of drying is attributable to the variety of physico-mechanical properties of different material types [17]. Wood composite materials exhibit sorption isotherms that differ from those of solid wood [11,13] caused by heat treatment during manufacturing [11,13]. Particleboard and medium-density fiberboard (MDF) exhibit a modified sorption curve [18]. Typically, they have 1% to 2% lower moisture content at a given temperature and humidity than solid wood [19].

### 2.2. Thermal Comfort

**Figure 2:**
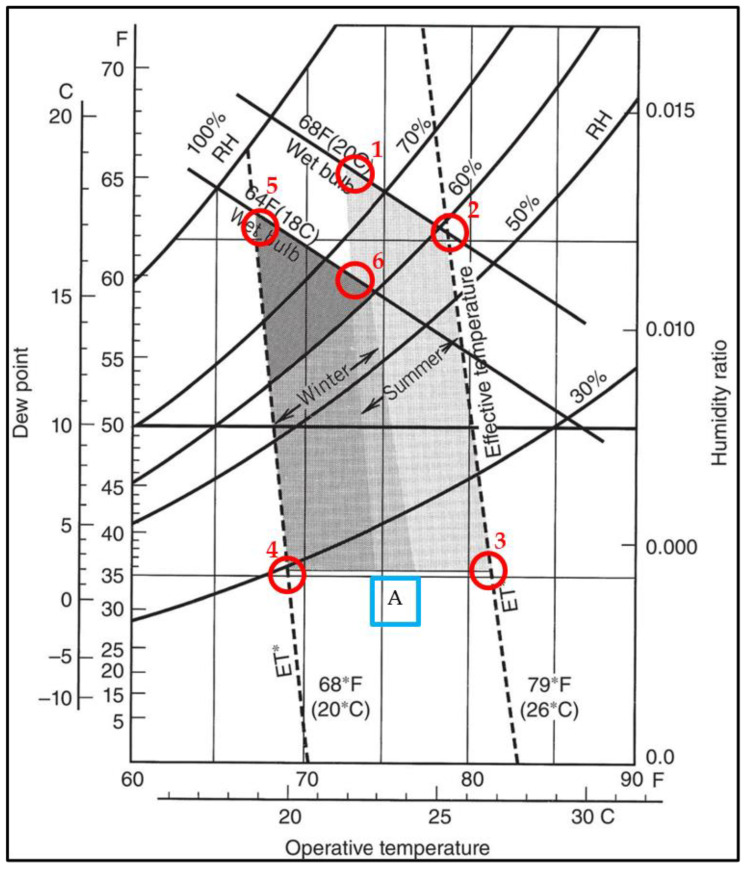
Acceptable operating temperature and humidity ranges for individuals wearing typical summer and winter clothing during light activity [19] (Copyright notice for ASHRAE Standards, ©ASHRAE, www.ashrae.org (accessed on 30 Novembre 2023). (1995) ASHRAE Standard (Addendum 55a-1995).

Thermal comfort is determined using guidelines based on research and standards. Most of this legislation refers to ANSI/ASHRAE SSa-1995 Standards [19]. These standards graphically represent the thermal environmental conditions suitable for human occupancy, detailing operative temperature and air humidity ratios for both winter and summer scenarios (Figure 2).

Six extreme points were chosen, representing the limits of the thermal comfort zone. Points 1-2-3 and A are the limits of the summer zone, and A-4-5 and 6 are the limits of the winter zone. An intermediate zone between the summer and winter zone is illustrated by A. All thermal comfort conditions are included in the area limited by the circled points 1-2-3-4-5 and 6 in Figure 2. These six extreme points constitute the study’s environmental conditions parameters for measuring the drying time in the thermal comfort zone.

### 2.3. Conceptual Formulation Overview

This section presents the principal and general formulas to provide a comprehensive foundation and clear understanding of the study’s principles.

#### 2.3.1. Moisture Content (MC)

Moisture content (MC) represents the amount of water in wood-based material expressed as a percentage of dry wood weight. It can be calculated using the following formula [9]:

(1) $$MC=\frac{m_{water}}{m_{wood}}\times 100\%,$$

Operationally, the moisture content of a given piece of wood can be calculated as follows:

(2) $$MC=\frac{m_{wet}-m_{dry}}{m_{dry}}\times 100\%$$

where *m_wet_* is the mass of the specimen at a given moisture content and *m_dry_* is the mass of the oven-dry specimen.

#### 2.3.2. Equilibrium Moisture Content (*EMC*)

Nelson (1983) developed a model based on Gibbs free energy to describe the sorption behavior of cellulosic materials. The model is of the form [20,21]:

(3) $$\frac{RH}{100}=\exp\left\{\left(-\frac{W_{w}}{R.T}\right)\exp\left[A\left(1.0-\frac{EMC}{M_{V}}\right)\right]\right\},$$

The inverse form of Equation (3) is:

(4) $$EMC=M_{V}\left\{1.0-\frac{1}{A}\ln\left[\left(-\frac{R.T}{W_{w}}\right)ln\left(\frac{RH}{100}\right)\right]\right\},$$

In these equations, *RH* is the relative humidity (%), *exp* is the exponential function, *W_W_* is the molecular weight of water (18 g·mol^−1^), *R* is the universal gas constant (8.314 J·K^−1^·mol^−1^), *A* is the natural logarithm (*ln*) of the Gibbs free energy as the relative humidity approaches zero, and *M_V_* is a material constant that approaches the fiber saturation point for desorption (*%*).

### 2.4. Determination of Test Parameters

The parameters for this study were derived from Figure 2, where absolute humidity was converted to relative humidity, followed by the application of Equation (4) for validation. Understanding the moisture distribution within wood-based materials under specific environmental conditions is crucial for setting accurate test parameters.

The moisture distribution over time depends on direct variables such as time (t) and distance (*x*). Also, it depends on the diffusion coefficient (*D*). The previous studies show that there is no direct algebraic formula to define the moisture distribution as a function of time and distance. This study utilizes a finite-difference solution of Fick’s second law to model moisture distribution:

(5) $$\frac{\partial M}{\partial t}=\frac{\partial }{\partial x}\left[D(M)\frac{\partial M}{\partial x}\right],$$

where *M* represents moisture distribution, *D(M)* is the diffusion coefficient as a function of *M*, *t* is time, and *x* is distance [13,21].

Equation (5) is derived from an approximation of Fick’s first law and Stamm’s equations, which can be solved graphically or algebraically. Both solutions assume isothermal conditions and may be resolved by transforming them into finite-difference equations, followed by iterative numerical solutions [21,22]. However, the discretization process must be carefully adapted for accuracy. Solving Equation (5) analytically is possible based on some assumptions, but it is a more complicated alternative [23].

For points within the thermal comfort boundaries that do not align with the moisture content (MC) setpoint range of 12% to 8%, the nearest value that does was selected. For instance, if a tendency point exceeds the 12% setpoint by 17.5%, it is adjusted to the nearest value within a ±0.5% range of the 12% setpoint. It was observed that conditions # 1 and # 5 exceeded the acceptable range (over 12%), necessitating adjustments to align with the desired MC range of 12% to 8%. These modifications are detailed in Table 1.

Correction is achieved by reducing the relative air humidity, as its effect on moisture variation is less significant than that of temperature. A direct relationship exists between relative air humidity and moisture; a decrease in relative air humidity leads to a corresponding decrease in moisture, and vice versa. Conditions #3 and #4 result in EMC values below the permissible limit of 8%. However, these conditions are still accepted, given that the drying process (desorption) progresses from higher to lower moisture levels. Furthermore, the initial saturated moisture value of the samples will exceed 12%, which will be explained later.

## 3. Theoretical Foundations and Mathematical Modelling

Having defined the critical problem of moisture management, this section builds upon the identified challenges by developing a theoretical mathematical model to describe moisture transfer phenomena in wood-based materials, paving the way for subsequent empirical validation and simulation analysis.

This study begins with a comprehensive review of the recent literature to identify the optimal solution. This will justify the methodological approach. Then, a customized mathematical solution tailored to our specific needs is developed and tested to evaluate its effectiveness.

### 3.1. Literature Review

In exploring moisture diffusion in wood-based materials, the literature reveals a division of methodologies into two primary categories: models based on potential approaches and multi-component models [24]. This distinction provides a foundation for understanding the diverse strategies employed to investigate moisture behavior within these materials.

Crank and Park [25] proposed a method to determine the diffusion coefficient D through experiments and mathematical techniques. Their experimental process involved observing weight changes (absorption rate) of a sheet with thickness “l” in a vapor atmosphere at constant temperature, pressure, and vapor concentration. The mathematical formula was derived using a first-order approximation. Building on this, Crank further developed an advanced formula [23] through a series of approximations. This formula is based on the boundary condition that the surface reaches immediate moisture content equilibrium with the surrounding atmosphere, allowing for the determination of the diffusion coefficient’s dependency on moisture content when half of the total sorption has occurred.

The description of Luikov [26] of heat and mass transfer in capillary-porous bodies highlighted the role of capillary forces and temperature gradients in moisture transport. This perspective was instrumental in shaping subsequent models intended for drying scenarios, particularly in the context of wood-based materials. Similarly, Simpson [27] initially focused on predicting the equilibrium moisture content of solid wood and comparing different theories. Simpson [28] later adopted an experimental approach to measure moisture absorption across various moisture contents, using numerical methods and finite differences to solve the diffusion equation. This analysis revealed that the diffusion coefficient increased with moisture content, particularly between 2.5% and 18.0% humidity. The collaborative efforts of Simpson and J.Y. Liu [29] further advanced the understanding of moisture diffusion, revealing an exponential increase in the diffusion coefficient with moisture content. The authors developed an equation to separate the diffusion coefficient D from the surface emission coefficient S, facilitating numerical methods for cases where surface equilibrium is not immediate. This method significantly reduced the experimental effort required. Simpson and J.Y. Liu [30] discussed the water diffusion coefficient’s dependence on water content in aspen *(Populus spec.).* They elaborated on mathematical techniques used by Crank and Park (1949) and reanalyzed Simpson’s (1974) experimental data. Their findings indicated an exponential increase in the diffusion coefficient with moisture content.

Based on transport equations in continuous media, Whitaker [31] approached drying as a transport phenomenon of heat and mass in porous media, considering the movement of water through a porous medium, gradients in total pressure, and energy balance. However, the study uses a multi-component approach that is established on assumptions that lead to several limitations. This challenge set the stage for Siau [22], which presents concepts related to wood–environment interactions, emphasizing the non-isothermal transport of bound water. The study views diffusion as a flux of molecular mass influenced by concentration gradients and water potential, relying on the methodologies Crank (1956) developed for calculating diffusivity.

Further expanding on these concepts, Stanish et al. [32] combined mathematical modeling and experiments to simulate drying in hygroscopic porous media. They developed a mathematical model to identify significant transport modes, demonstrating agreement between model predictions and experimental results. Y. Fortin [33] further explored wood’s water content and water flow properties, particularly at high moisture content. Fortin described the relationship between water content and flow properties as exponential, potentially temperature-dependent, and related to water potential. Building on this concept, A. Cloutier and Y. Fortin [34] applied water potential concepts to wood–water relationships and combined them with experimental techniques. They used two methods to establish the relationship across the entire moisture content range, discovering that water potential increased with temperature.

In 1993, A. Cloutier and Y. Fortin [24] published an article proposing a model for moisture movement in wood during isothermal drying. This model employed water potential gradients as driving forces and effective water conductivity as the moisture transport coefficient. The results confirmed the model’s validity.

The inquiry into moisture movement was extended to medium-density fiberboard (MDF) panels by S. Ganev and A. Cloutier [21], who examined the effects of density and sorption state on sorption isotherms and diffusion coefficients. Their findings highlighted the critical influence of moisture content on water conductivity, a crucial insight for understanding moisture behavior in composite wood materials. Building on this foundation, L. Cai and S. Deku [35] explored moisture transfer in particle boards using the finite element method (FEM) and linear regression. Their experiments demonstrated that diffusion occurred both perpendicular and parallel to the panel surface, with orientation affecting diffusion coefficients.

Building upon the foundational work in the field, Q. Wu and O. Suchsland [13] studied diffusion in overlapping panels, including particleboards, particleboards overlaid with high-pressure laminated panels (HPL), and particle boards with HPL front and back (HPL backer). They examined sorption behavior and moisture distribution in different boards as relative air humidity varied. Further advancing this line of inquiry, Q. Wu [12] applied Nelson’s formula to determine sorption isotherms for various wood-based materials, confirming its applicability. Experimental procedures were established by taking samples of the materials of oriented strand board (OSB), particle board, medium density fiberboard (MDF), hard board (HB), high-pressure laminate board (HPL and HPL backer), and wood (Pinus). In a subsequent study, Q. Wu and M. Xiong [36] proposed a simple linear empirical model for simulating moisture diffusion in MDF boards used for furniture. This model related the diffusion coefficient, position, and vapor pressure in a single equation.

Table 2 summarizes these methods while providing a comprehensive summary of the formulas, strengths, and limitations identified in the literature, offering a clear overview of the state of research in moisture diffusion modeling for wood-based materials.

### 3.2. Development of the Mathematical Model

The developed mathematical model is based on the work of Q. Wu and M. Xiong [36], who define the diffusion coefficient *K(p*, *x)* as follows:

(6) $$K\left(p,x\right)=0.000431-0.00107p+0.00192x+0.0005p^{2}-0.00192x^{2}+0.000206.x.p.,$$

The relative air humidity (RH) can be expressed as the ratio of the partial vapor pressure in the air to the saturation vapor pressure in percent [22].

(7) $$RH=\frac{P}{P_{sat}},$$

(8) $$P=RH\times P_{sat},$$

On the other hand, Monteith and Unsworth [38] developed a new form of Teten’s formula for temperatures above 0 °C:

(9) $$P_{sat}=e_{s}\left(T\right)=0.611\exp\left(\frac{17.27\;(T-273)}{T-36}\right)\;(Kpa),$$

From Equations (8) and (9), the pressure P is defined by

(10) $$P\left(RH,T\right)=RH\times 0.611\exp\left(\frac{17.27\;\left(T-273\right)}{T-36}\right)\left(Kpa\right),$$

From (6) and (10), diffusion *D* can be expressed as a function of relative humidity *RH*, temperature *T*, and position x as below:

(11) $$\;\;\;\;\;\;\;\;\;\;K\;\left(RH,T,x\right)=0.000431-0.00107\left(RH\times 0.611\exp\left(\frac{17.27\;\left(T-273\right)}{T-36}\right)\right)\;\;\;\;\;\;\;\;\;\;\;\;\;\;\;\;\;\;+0.00192x+0.0005{\left(RH\times 0.611\exp\left(\frac{17.27\;\left(T-273\right)}{T-36}\right)\right)}^{2}\;-0.00192x^{2}\;\;\;\;\;\;\;\;\;\;\;\;\;\;\;\;\;+0.000206.x.\left(RH\times 0.611\exp\left(\frac{17.27\;\left(T-273\right)}{T-36}\right)\right),$$

However, the diffusion coefficient *K* in the formula of *Q*. Wu and M. Xiong [36] is in (g/cm·mmHg·h). At the same time, dimensional analysis is required in most books and simulation software that use the international system MKSA. Dimensional analysis (equations to dimensions) allows us to validate the homogeneity of the formula by its units.

(12) $$D=\frac{L^{2}}{T}\;and\;K=\frac{M}{L\times P\times T},$$

(13) $$\frac{D}{K}=\frac{L\times P\times T}{M}\times \frac{L^{2}}{T}=\frac{L^{3}\times P}{M},$$

To homogenize the equation, a dimensionless coefficient α is provided to be determined subsequently. In this case, the formula will be as follows:

(14) $$\frac{D}{K}=\;\propto \times \frac{L^{3}\times P}{M},$$

*P* is the pressure unit in Pascal (Pa), *L* is the unit of length in meters (m), and *M* is the mass unit (kg). The *L*^3^/*M* ratio represents *1/ρ* considered constant.

$$K=\left[\frac{g}{cm\times mmHg\times h}\right]=\left[\frac{0.001\;kg}{0.1\;m\times 133.32\;Pa\times 3600\;s}\right]\;\;\;\;=2.084\times 10^{-8}\left[\frac{kg}{m\times Pa\times s}\right]$$

$$\propto =2.084\times 10^{-8},$$

$$D=2.084\times 10^{-8}\times \frac{P}{\rho }\;K,$$

The final form of Equation (11) will be:

(15) $$D\;\left(RH,T,x\right)=2.084\times 10^{-8}\times \frac{RH\times 611\;exp\left(\frac{17.27\;\left(T-273\right)}{T-36}\right)}{\rho }\;\;\;\;\;\;\;\;\;\;\;\;\;\;\times \left[0.000431-0.00107\left(RH\times 611\exp\left(\frac{17.27\;(T-273)}{T-36}\right)\right)\;\;\;\;\;\;\;\;\;\;\;\;\;+0.00192x+0.0005{\left(RH\times 611\exp\left(\frac{17.27\;(T-273)}{T-36}\right)\right)}^{2}\;\;\;\;\;\;\;\;\;\;\;\;\;\;\;\;-0.00192x^{2}+0.000206.x.\left(RH\times 611\exp\left(\frac{17.27\;(T-273)}{T-36}\right)\right)\right],$$

Subsequently, Equation (15) will be adapted and used in the simulation considering the conditions of our study, namely, the other input parameters, the wood-based materials (melamine and MDF), and the geometry of the samples.

## 4. Numerical Simulation and Analysis

With the theoretical model established, numerical simulation and analysis apply these foundational concepts to visualize moisture behavior under varying conditions. These simulations are instrumental in validating our theoretical assumptions and refining the ANN model’s predictions, thus contributing significantly to our research objectives.

### 4.1. General Description

Simultaneous heat and mass transfer through the porous medium of wood-based materials play a crucial role in reducing moisture content (MC) and facilitating their transformation [39]. To simulate these phenomena, a Multiphysics model was developed using COMSOL software (COMSOL Multiphysics v6.0), which models coupled heat and moisture transport in porous media (ham) based on the finite element method (FEM). This model effectively describes the moisture transfer in melamine and MDF. However, it is necessary to adjust specific parameters to better align with the actual case. These adjustments include parameters dependent on temperature and material properties, such as diffusivity, porosity, and density. The choice of this software was motivated by its simplicity and practical capability to couple heat and mass transfer models in wood-based materials [6].

### 4.2. Governing Equations

Wood-based materials, like many porous media, require specialized modeling in software like COMSOL Multiphysics (V. 6.0). However, parameter adjustments are necessary to represent real-world conditions accurately. The governing equations are derived under several key assumptions:

(1) The mass transfer of moisture occurs in vapor form.
(2) Drying is merely driven by natural heat and mass transfer (natural drying).
(3) The diffusion is based on an approximative equation.

“Heat transfer in porous media” and “Moisture transport in porous media” were the focus areas.

#### 4.2.1. Heat Transfer in Porous Media

Porous media are characterized by moisture movement from within the material to the surrounding air. Heat transfer is used to increase the temperature of the product, which leads to a thermodynamic state that favors moisture transfer in porous media. Heat transfer in moist air is determined as follows:

(16) $${\left({\rho C}_{p}\right)}_{eff}\frac{\partial T}{\partial t}+{\rho C}_{p\;}u\;\cdot \nabla T+\nabla q=Q,$$

(17) $$q=-k_{eff}\nabla T,$$

#### 4.2.2. Moisture Transport in Porous Media (Diffusion Model)

In our case, the moisture transport is modeled considering the vapor transport flux as the sole means of water transport. However, temperature and relative humidity significantly influence wood-based materials’ final moisture content and drying time. Moisture transfer is described using the following equations:

(18) $$\frac{\partial w(\emptyset_{w})}{\partial t}+{\rho_{g}u}_{g\;}\cdot \nabla \omega_{v}+\nabla g_{w}=G,$$

(19) $$w\left(\emptyset_{w}\right)=\varepsilon_{p}s_{g}\rho_{g}\omega_{v},$$

(20) $$\omega_{v}=\frac{M_{v}\emptyset_{w}c_{sat}}{\rho_{g}},$$

(21) $$g_{w}=\rho_{g}D_{eff}\nabla \omega_{v},$$

#### 4.2.3. Input Parameters

Table 3 provides details of the model’s parameters used in the simulation:

### 4.3. Geometry and Mesh Generation

**Figure 3:**
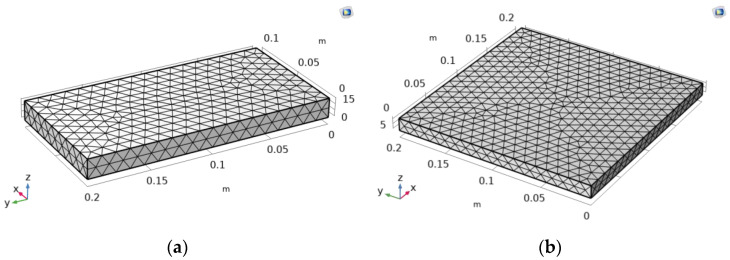
Mesh of the model to be simulated: (**a**) melamine, (**b**) MDF.

The selected geometry is a 3D rectangular parallelepiped, measuring 200 × 100 × 15.8 mm^3^ for melamine and 200 mm × 200 mm × 12.7 mm for MDF. This geometry is consistently applied across all study sections to facilitate the comparison of results. The predefined mesh, featuring a free tetrahedral geometry, is automatically generated by COMSOL (Figure 3).

An optimal balance between precision and calculation speed is aimed to be achieved through the selection of mesh size, as investigated in a mesh sensitivity study. This is carried out by selecting a point at the center, where moisture content values obtained from Equations (4) and (15) are compared with those from the simulation. The error, calculated as the difference between these values resulting from each mesh simulation, helps guide our choice. The parameters from Table 3 were employed for the initial and boundary conditions, and simulation was conducted until stability was reached after 120 h. Variations in mesh size yield error values and computation times, as detailed in Table 4.

**Figure 4:**
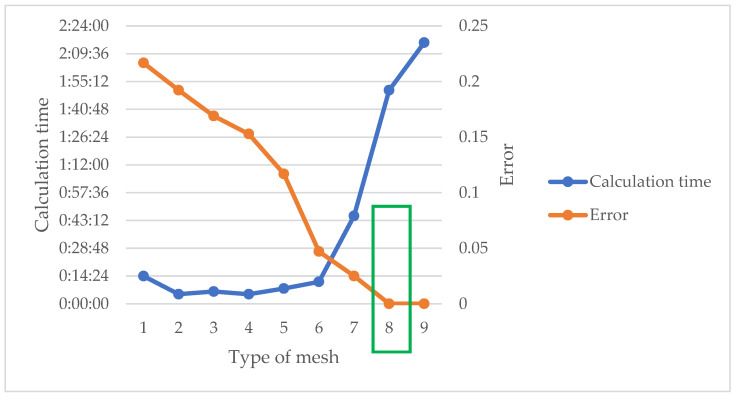
Graph illustrating mesh sensitivity study. The green border represents the adopted mesh.

Figure 4 illustrates the error and calculation time variations according to each mesh.

Table 4 and Figure 4 were analyzed, leading to the selection of the mesh that best compromises between precision and calculation speed. The choice of mesh type was N° 8, which corresponds to the mesh “Finer” in COMSOL.

### 4.4. Simulation Results and Discussion

**Figure 5:**
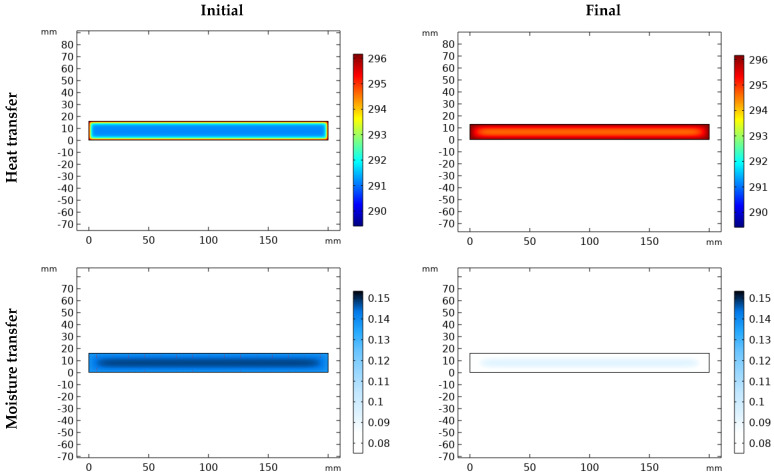
Heat and mass transfer in melamine.

**Figure 6:**
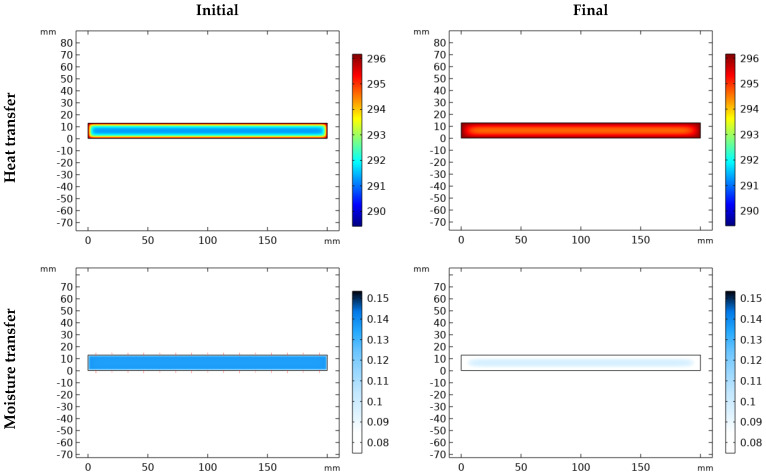
Heat and mass transfer in MDF.

**Figure 7:**
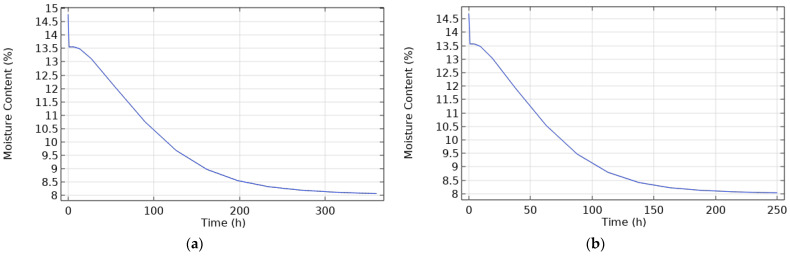
Moisture content evolution in time using simulation solution for (**a**) melamine, (**b**) MDF.

The simulation results are presented in two forms:

(1) Heat and moisture distribution: To show the transfer phenomena inside the material, the cross-section of the samples is presented according to the width and thickness. Figure 5 and Figure 6 show the evolution of combined heat and mass transfer in melamine and MDF, respectively. The heat transfer illustrates the temperature distribution and its evolution over time, from 0 to 2 h (depending on the condition), until it reaches a steady state. Notably, MDF reaches this state more rapidly than melamine due to the differences in their heat capacities within the temperature range of 292.15 °K and 300.15 °K (CP (melamine) > CP (MDF)). Furthermore, the boundaries of the specimen are influenced before the core. Moisture transfer occurs faster in MDF than in melamine and requires more time than heat transfer alone. When moisture transfer is combined with heat transfer, the drying process accelerates significantly compared to moisture transfer alone (more than 240 h). Therefore, temperature emerges as the pivotal factor in drying, particularly in drying time.

(2) Moisture content Graph: The simulation provides a detailed view of the phenomenon. Figure 7a,b illustrate the drying process for condition (5) (Table 1), divided into two stages. The first stage is characterized by a rapid drop in moisture content within a short period (2 h). This phenomenon results from the combined effects of heat and moisture transfer. The second stage features a gradual decrease in moisture content as the temperature stabilizes throughout the specimen until it reaches the equilibrium moisture content (EMC) or the desired values. The overall curve shape is consistent across all conditions, with differences primarily observed in the duration of each stage and the final moisture content. A comprehensive comparison of all graphs is presented in Section 7.2.

The simulation results, particularly from points (1) and (2), emphasize the following aspects:

- The factors influencing the simulation are associated with the materials (such as the particles’ heterogeneity, nature, or size, and additives), the approximation equations, and the phenomenon’s complexity.
- Simulation allows us to visualize moisture content evolution within the materials and identify the stages of the phenomenon.
- Temperature plays the primary role in drying, especially for drying time.
- Like most wood-based materials, melamine and MDF exhibit comparable general behavior in response to their environment.

## 5. Experimental Study

Reflecting on the Introduction, which highlighted the critical role of optimizing moisture content for energy efficiency and sustainability in wood product manufacturing, this section details the experimental study conducted on melamine and MDF under specified thermal comfort conditions. The experimental investigation is crucial for validating the theoretical models and numerical simulations previously discussed, providing empirical data to fine-tune the ANN model. By precisely tracking moisture content evolution in real-world scenarios, this segment directly contributes to fulfilling our study’s aim of optimizing drying processes, thereby enhancing energy efficiency and sustainability in wood product manufacturing.

### 5.1. Methodology of the Experimental Study

Initially, our knowledge of the materials was limited. Consequently, the determination of the properties of the dehydrated and humidity-saturated samples was necessary. The behavior of wood-based material samples under thermal comfort conditions was then studied. Accordingly, the experimental approach adopted follows these three steps:

1. Step 1: Drying until obtaining the anhydride mass, m_dry._ All samples were placed in a kiln dryer at 80 °C and measured their weight daily using a precision balance until it became stable (no weight variation). The final weight represented the anhydride mass m_dry_ (0% moisture).
2. Step 2: Attaining maximum moisture level (saturated moisture). After the anhydrite samples were obtained, they were placed in a conditioning chamber under extreme humidity conditions (23 °C, 95%) and obtained the maximum wetted mass m_wet_ by measuring the weight until it became stable. This step allowed us to determine the maximum wetted mass (saturated moisture).
3. Step 3: Applying test parameters. The final step involved applying the test parameters outlined in Table 1 and calculating the time required to reach 12% or 8% moisture content levels. During this step, moisture content variations were measured over time. This experience aimed to establish the necessary drying time for each sample.

Weight is measured with a precision scale in each step, and the MC is calculated using Equation (2). Then, in the third step, two samples for each condition are provided to calculate the average MC for more precision. The samples are identified according to the form iXj, where

- i: Test number (1 to 2). For the average, the letter M is used.
- X: Material (M for MDF, P for melamine).
- j: Number of test conditions (1 to 6).

### 5.2. Test Devices

The experiments are conducted using the following devices:

- Kiln dryer: A kiln dryer assures the experience parameters of the first step at 80 °C.
- Weighing (precision balance): The precision balance measures the weight accurately, with a precision of 0.01 g and a maximum capacity of 1000 g.
- Conditioning chamber: The conditioning chamber ensures the experience parameters of the second and third steps with real-time control.

### 5.3. Materials

**Figure 8:**
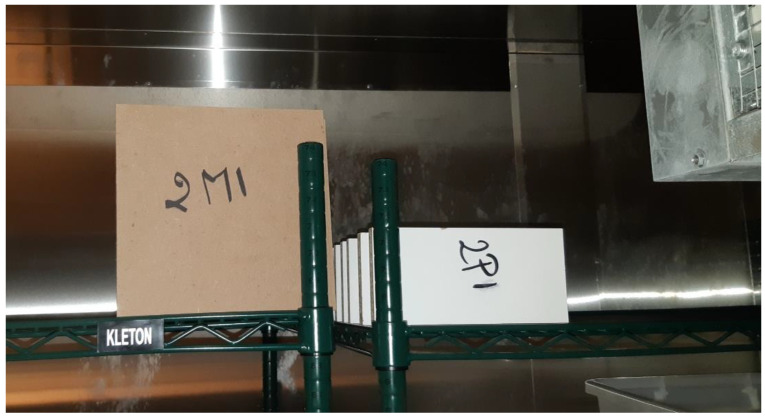
Test samples (M for MDF and P for melamine).

Test samples were taken from commercially available materials: melamine and MDF (Figure 8). As summarized in Table 5, two specimens are used for each condition to calculate the average.

1. **Melamine:**
  - The test samples were 200 mm (length) × 100 mm (width) × 15.8 mm (thickness);
  - Samples number: 12 (1P1, 1P2, 1P3, 1P4, 1P5, 1P6, 2P1, 2P2, 2P3, 2P4, 2P5, 2P6);
  - Density: 728 kg/m^3^ (calculated).
2. **MDF:**
  - The test samples were 200 mm (length) × 200 mm (width) × 12.7 mm (thickness);
  - Samples number:12 (1M1, 1M2, 1M3, 1M4, 1M5, 1M6, 2M1, 2M2, 2M3, 2M4, 2M5, 2M6);
  - Density: 728 kg/m^3^ (calculated).

### 5.4. Experimental Results and Discussion

Step 1: Drying to 0%—All samples.

Initially, the moisture levels of the samples were unknown and could vary. To establish a reliable starting point for our experiments, they were dried to achieve 0% moisture using a kiln dryer at 80 °C. Drying continued until the weight became stable. Table 6 provides the anhydrous sample weights (MC = 0%).

Step 2: Reaching maximum moisture—All samples.

**Figure 9:**
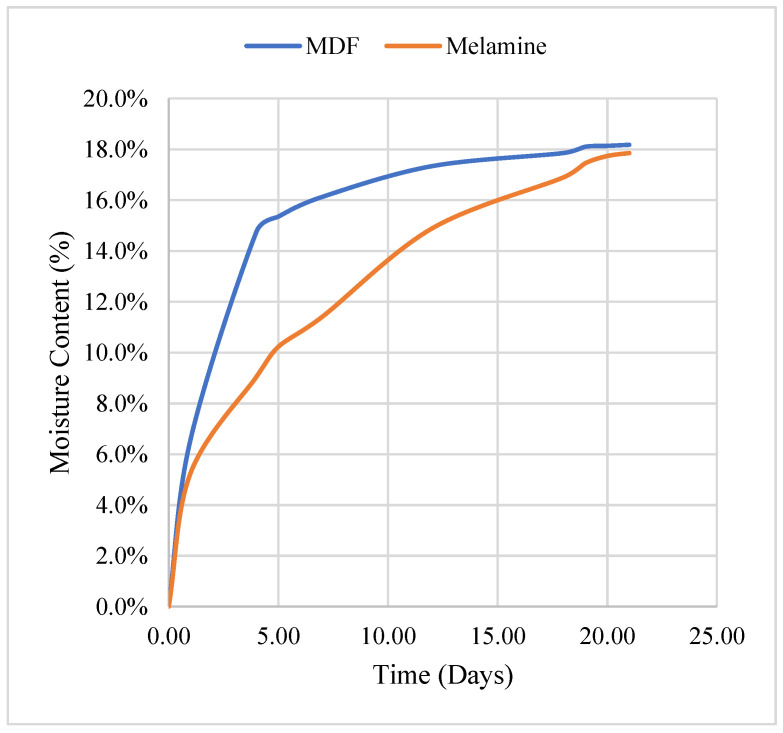
Evolution of moisture under extreme conditions (23 °C and 95%) for melamine and MDF.

Subjecting the samples to extreme conditions (23 °C and 95% humidity) enabled us to determine the maximum moisture levels for melamine and MDF and the time required to reach a stable state. Table 7 provides the values of MC for melamine and MDF under extreme conditions during the test period. After that, the MC remained stable. Figure 9 shows this evolution in time.

Step 3: Applying test conditions (Table 1)

**Figure 10:**
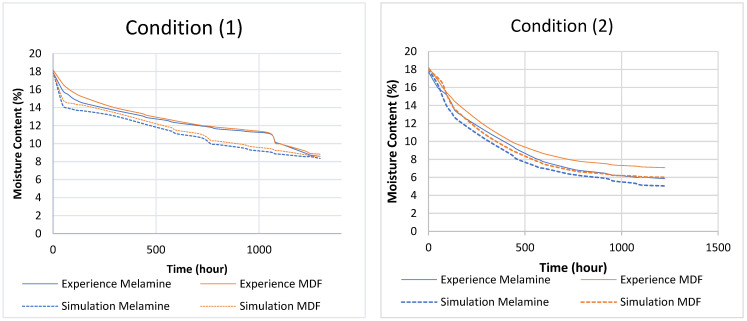

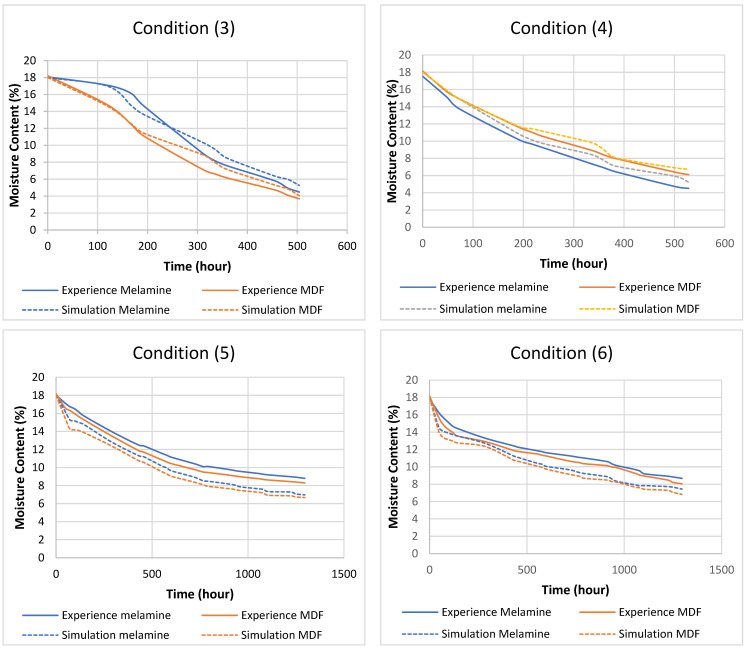
Comparison between the experimental results and the simulation: (1) 23 °C and 65%; (2) 26 °C and 57%; (3) 27 °C and 20%; (4) 20 °C and 31%; (5) 19 °C and 65%; (6) 23 °C and 63%.

In this step, the test conditions identified in the six points of Table 1 are applied, as illustrated in Figure 3. These tests illustrate the moisture evolution in time for melamine and MDF and the required delay for each condition to reach the admissible MC, between 8% and 12%. The results for the six test conditions are presented in Table 8, and the corresponding graphs will be provided in the comparison section (Figure 10) *.

* N.B: to avoid repetition, the graphs will be presented in the comparison section in Figure 10.

The results demonstrated that it is possible to dry all the samples in ambient conditions to the setpoint zone limited by 12% and 8% MC. However, differences occur in the time required for the sample to reach or pass this zone, i.e., the drying time, according to the material and environmental conditions.

Upon the analysis of the general form of figures (see Figure 10) and numerical values, it was observed that:

- Drying (desorption) and wetting (adsorption) are inverse processes.
- The graphs present a pseudo-exponential form.
- The sample exposed to higher temperatures dried faster.
- The sample exposed to lower air humidity also dried faster.
- MDF dries, under most conditions, faster than melamine.

## 6. ANN Design and Implementation

Building on the foundational concepts outlined in the introduction, this section introduces the practical application of the theoretical and empirical findings through artificial neural networks (ANN). This section underscores our approach to precisely predicting moisture content variations in wood-based materials, leveraging ANN to address the complex interactions between environmental conditions and material responses. In this section, the design and implementation process of the ANN models is detailed, illustrating their role in achieving the research objectives of enhancing moisture management and operational efficiency in the furniture manufacturing industry.

### 6.1. General Description

The first challenge is transforming the phenomena of moisture transfer in wood-based materials into a mathematical model [22]. One approach employs numerical simulation to address the physical transfer phenomena occurring during drying. However, in this study, we opt for a heuristic artificial neural network (ANN) approach to model these phenomena, using parameters derived from experimental results. Researchers have increasingly employed ANN to optimize wood-based material drying due to its speed, adaptability, and general applicability compared to traditional methods [22,23]. Our ANN methodology comprises three steps:

1. Firstly, determine the ANN architecture.
2. Secondly, train the model, and validate its final configuration by calculating the mean squared error (MSE) and the coefficient of regression R.
3. Thirdly, once the neural network predicts the time required to reach moisture content (MC) levels of 12% and 8%, compare these predictions with the experimental results by calculating the errors.

Given the complexity of the phenomena, a separate ANN configuration was developed for each material: melamine and MDF.

### 6.2. Model Building

Developing the ANN model involves establishing the architecture, input–output parameters, training algorithm, and performance indices. The selection of architecture depends on the specific phenomena under study. Referring to previous studies [22,24,25], most ANN architectures can yield good results, especially in prediction problems. The backpropagation (BP) method, coupled with the Levenberg–Marquardt training algorithm, is well suited for this study due to its simplicity and speed. Transfer functions used include the hyperbolic tangent function and linear function.

**Figure 11:**
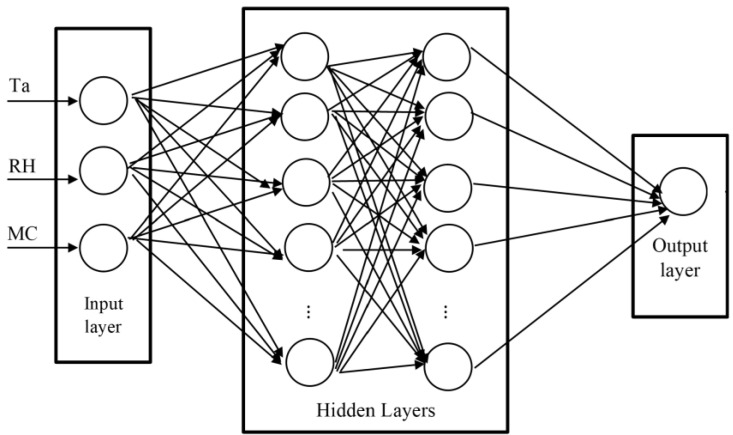
Proposed ANN architecture.

Input parameters comprise air temperature (Ta), relative humidity (RH), and target moisture content (MC), while the output sought is drying time (td). Consequently, the ANN network adopts a general form of 3 − n(i, j) − 1 (i: hidden layers number, j: number of neurons in each hidden layer) (Figure 11). The specific values for i and j will be determined in the results section.

### 6.3. Performance Parameters

Determining the performance parameters of the ANN model is performed by calculating the coefficient of regression R and mean square error MSE. The correlation coefficient can be calculated from the following equation:

(22) $$R^{2}=1-\frac{\sum_{i=1}^{n}{\left(y_{i}-{\hat{y}}_{i}\right)}^{2}}{\sum_{i=1}^{n}{\left(y_{i}-{\bar{y}}_{i}\right)}^{2}},$$

**Figure 12:**
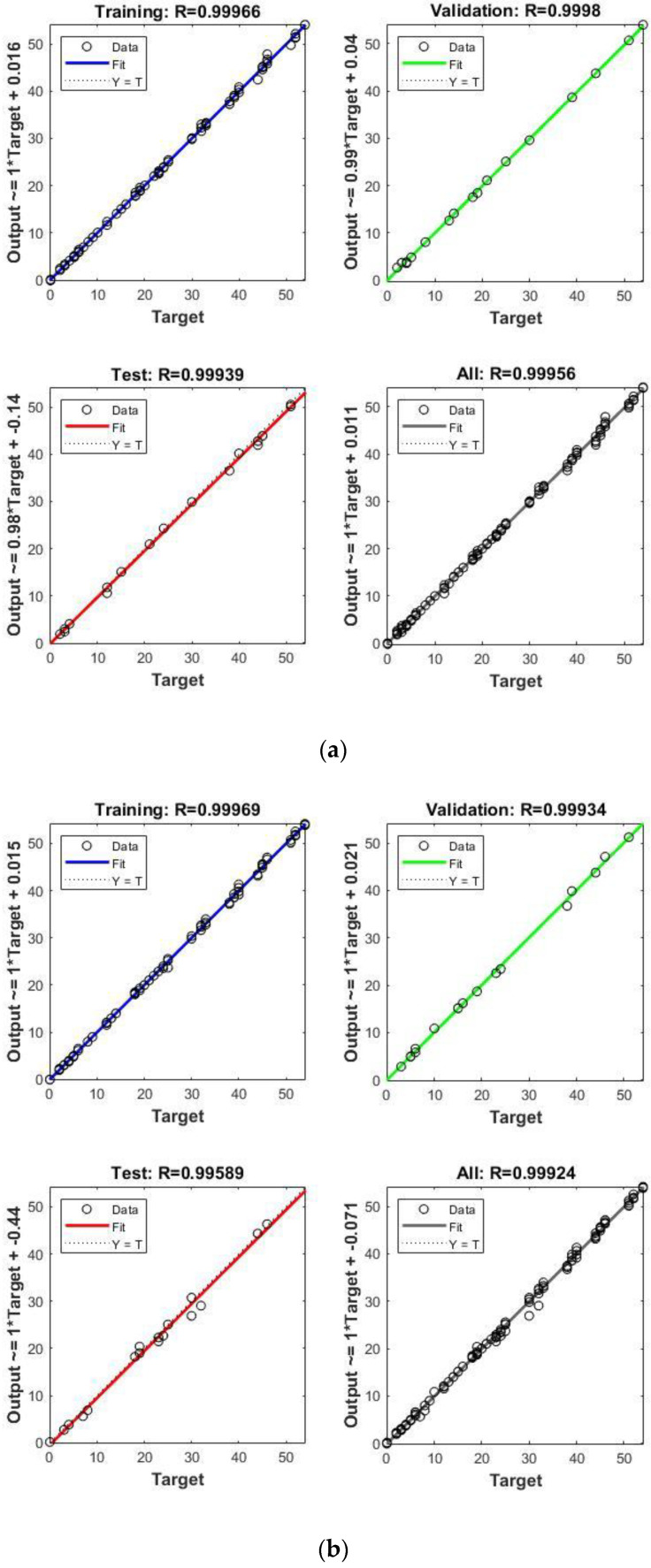
Correlation coefficient variation for (**a**) melamine and (**b**) MDF.

It measures the variation in the fitted model and compares models by assessing which one provides the best fit to the data. The resulting variation in the correlation coefficient for melamine and MDF is shown in Figure 12.

The MSE measures the average deviation between the fitted values and the actual data observation [26]. The MSE equation is as follows:

(23) $$MSE=\frac{\sum_{i=1}^{n}{\left(y_{i}-{\hat{y}}_{i}\right)}^{2}}{n},$$

MATLAB software offers an integrated tool called Network Toolbox. This tool allows the user to build, train, and test the network and computes R and MSE.

### 6.4. ANN Results and Discussion

To determine the final network architecture, the indices of hidden layers (i and j) were varied, and the input–output parameters were tested, where i is the hidden layers number and j is the neuron number in each hidden layer. Finally, the ANN values are compared to the measured value for each combination, and the R and MSE are calculated using Equations (22) and (23). The experimental data are divided into three sets: 70% is used for training, 15% for validation, and 15% for testing the model.

The final configuration considers the performance parameter MSE and regression R, as well as the response time (as short as possible).

**Figure 13:**
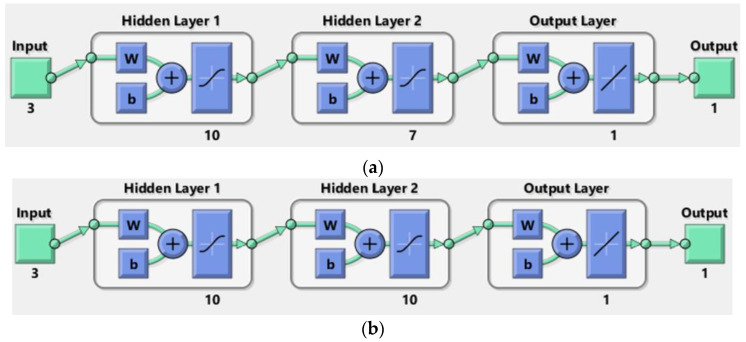
ANN architecture for (**a**) melamine and (**b**) MDF.

**Figure 14:**
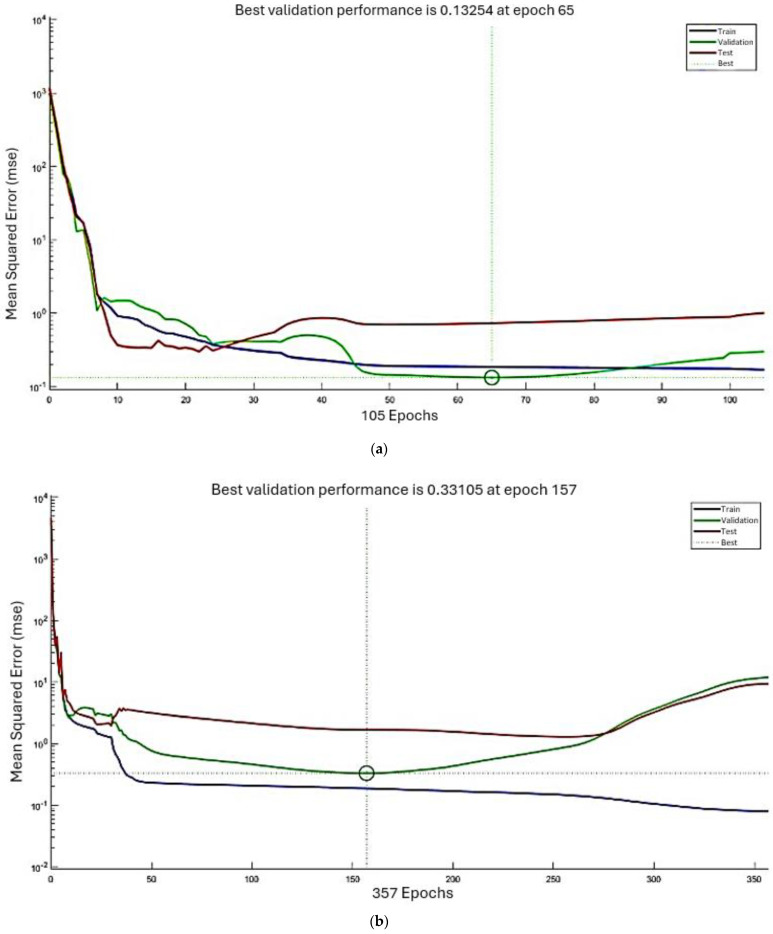
MSE variation for (**a**) melamine and (**b**) MDF.

The numerical results are presented in Table 9, and the corresponding diagrams are shown in Figure 11, Figure 13 and Figure 14. The best-fitted configuration is determined to be 3-10-7-1 for melamine and 3-10-10-1 for MDF.

Based on the above results, we conclude that the ANN model accurately represents the variation in moisture content (MC) and drying time. The mean squared error (MSE) and the coefficient of regression R are used to measure the ANN performance. The general value of R for melamine is 0.99956, and the MSE for the ANN training is 0.17. For MDF, the general value of R is 0.99924, and MSE for ANN training is 0.08, signifying that both melamine and MDF neural networks have an acceptable convergence.

## 7. Comparative Analysis

### 7.1. Comparison Criterion

The comparison criterion is the percent error calculation (PE). The percentage error (PE) allows us to evaluate the magnitude of the difference between the experimental values and simulation results or the experimental values and the ANN results.

(24) $$PE=\frac{\left|Mesured\;value-Real\;value\right|}{Real\;value}\times 100\%,$$

### 7.2. Experience vs. Simulation

The comparison between the experimental results and the simulation consists first of illustrating the experimental results and the values resulting from the simulation in the form of graphs and comparing their appearance. The graphs in Figure 10 show the evolution of the moisture content of melamine and MDF as a function of time for each condition. Then, an error calculation was performed.

The error is calculated throughout the humidity rate by taking a fixed time value. Then, for each time value, the relative error is calculated, and the minimum value, maximum value, and average of the errors for each condition are deduced. The numerical results of the comparison are presented in Table 10.

The maximum observed error is 25.4% while the minimum is 0.2%. The graphs show a similarity between the experiment and the simulation. The average error varies between 4.4% and 14.2%. These results are generally acceptable, but interpretation is needed to understand the source of the more significant discrepancies.

### 7.3. Experience vs. ANN

The comparison between experimental and ANN models consists of determining the required time to reach the allowable MC values (8% and 12%) for each test condition and calculating the PE by applying Equation (24). The results are presented in Table 11. In addition, Table 11 gives the required time for each condition to reach the permissible MC values, i.e., 12% and 8%.

The result comparison consolidates our approaches and choices. The required time to reach the critical values of MC 8% and 12% for each test condition is determined and compared to the experimental results by calculating the percent error (PE). This procedure is completed for melamine and MDF. The results are presented in Table 11.

The comparison of ANN-predicted drying times with the remaining experimental results (15% reserved for model testing) involves calculating the percent error (PE) for each test condition. The average PE is 1.40% for 8% MC and 2.85% for 12% MC for melamine. For MDF, the average PE is 1.42% for 8% MC and 2.25% for 12% MC. Consequently, the ANN model demonstrates acceptable precision.

### 7.4. Interpretation of Comparison Results

The comparison between the experiment and the simulation is made through the analysis of Figure 10, which shows the evolution of the humidity rate as a function of time. The general shape indicates a similarity between experiment simulation graphs but with a difference in amplitude.

Whether observed or calculated, the error is related to three main factors: experimental error, simulation error, and errors inherent to the phenomenon itself. Experimental error results from reading precision, device accuracy, parameter fluctuations, methodology, and the number of tests.

The simulation may also introduce errors, beginning with initial assumptions like material homogeneity and parameter stability, extending through the approximate mathematical model and mesh sensitivity, and ending in the computational tools’ performance. Finally, the error caused by the phenomenon itself comes down to the overall understanding of the phenomenon and its non-linearity.

Comparison results reveal that errors are more significant at relatively high temperatures (27 °C and 26 °C in conditions 2 and 3) than at lower temperatures. At lower relative humidity (20% and 31% in conditions 3 and 4), more significant errors are observed compared to higher humidity levels. This observation can be attributed to two factors. Firstly, the simulation’s approximate mathematical model describes the phenomenon within a limited range, assuming vapor-state mass transfer. In contrast, experiments involve measurements and samples with water mass transfer in other states. Secondly, the simulation explicitly describes heat transfer, which occurs in a few hours depending on temperature, while moisture transfer takes longer. This combination of two phenomena and their time scales is more significant in the simulation than in the experiment results.

In comparing the experimental data and the ANN, the error is minimal and justifiable, as the ANN has been trained, validated, and tested with experimental data. The error is related to the ANN’s precision, and the performance indices determined in section §6.4 are validated by this error computation.

### 7.5. Comparative Analysis with Previous Studies

This study’s ANN-based predictive model for moisture content in wood-based materials demonstrates significant alignment with the methodologies and findings of earlier research while also introducing new computational efficiencies. The models presented in this study for melamine and MDF, with mean squared error (MSE) values of 0.17 and 0.08, respectively, and high correlation coefficients (R), underscore a substantial improvement in precision over some of the foundational models referenced in the literature review. For instance, Crank and Park’s seminal work [25], while pioneering in establishing methods to determine the diffusion coefficient D, did not cater to the non-linearities inherent in moisture diffusion in wood-based materials that the ANN models adeptly capture. Similarly, while Luikov’s [26] description of heat and mass transfer in capillary-porous bodies set the stage for understanding the complexities of the drying process, this study extends these concepts by implementing them into a predictive ANN framework that adapts to a range of environmental conditions. Moreover, Simpson’s [27] investigations into equilibrium moisture content and the subsequent work with J.Y. Liu [30] that illuminated the dependence of the water vapor diffusion coefficient on moisture content in aspen provide a backdrop against which the actual ANN model’s predictive capabilities can be contrasted. The models in this study exhibit the capability to accurately predict moisture content across a broader range of conditions, which is especially valuable for industrial applications where operational parameters often fluctuate.

By integrating ANN with a thorough experimental approach, we not only corroborate the findings of these past studies but also enhance the predictive framework, achieving precision and adaptability that contribute to more efficient and sustainable practices in wood product manufacturing. The results of this study, with an average error margin impressively low, at 1.40% for MC 8% and 2.85% for MC 12% for melamine and 1.42% for MC 8% and 2.25% for MC 12% for MDF, stand as a testament to the efficacy of ANN in this domain of study.

## 8. Conclusions

Managing moisture content in wood is crucial for the industry, with an optimal range of 8% to 12% recommended for various transformations. However, maintaining specific workshop thermal comfort conditions, such as a temperature of 21 °C and relative humidity of 35% to 45%, is often impractical. This study draws on a range of approaches to comprehensively investigate the thermo-hydric behavior of wood-based materials during drying under specific thermal comfort conditions.

In the first approach, a new mathematical model for diffusion was formulated, accounting for temperature and air relative humidity. This formula will be applied in future simulations, offering a more precise understanding of wood moisture behavior.

The second approach involved the development of a simplified three-dimensional model using COMSOL software. While this model accurately describes moisture transfer for minor temperature differences and microscopic scales, it has limitations for more significant temperature discrepancies or macroscopic scales.

Our experimental approach involved three critical steps: initial sample preparation, controlled drying and moisture saturation, and rigorous testing under simulated industry conditions—ensuring that our findings are both precise and highly relevant to real-world manufacturing practices in the wood industry. Conditions representing thermal comfort extremes are applied to the samples by harmonizing them through initial drying and moisture saturation, ultimately converging within the 8% to 12% moisture zone. The convergence rates vary based on temperature and air humidity, with MDF showing faster convergence than melamine.

In the fourth approach, we constructed an ANN model to predict and generalize moisture content within the thermal comfort zone. The ANN networks demonstrate acceptable convergence, characterized by configurations of 3-10-7-1 for melamine and 3-10-10-1 for MDF. The mean squared error (MSE) and coefficient of regression R indicate this convergence. We compared ANN and simulation predictions with experimental data for MC 8% and 12% to validate our results, calculating percent errors (PE). On average, errors are 1.40% for MC 8%, 2.85% for MC 12% for melamine, 1.42% for MC 8%, and 2.25% for MC 12% for MDF. These results are notably close, falling within acceptable error margins.

The differences in outcomes across approaches (experimental vs. simulation and experimental vs. ANN) are attributed to inherent methodological variations, assumptions, and the stability of material properties and conditions. Although each approach is precise within the scope of its initial assumptions and tools, our error analysis and comparisons show that both methods are complementary and suitable within their respective contexts.

This research study contributes to a deeper understanding of the thermo-hydric behavior of wood-based materials, offering valuable insights that can enhance processes and product quality in industries where moisture content is a critical factor, including woodworking and manufacturing.

## Figures and Tables

**Table 1:** Adopted Parameters.

Test #	Initial Test Conditions	Initial EMC Melamine (%)	Initial EMC MDF (%)	Corrected Test Conditions	EMC Melamine (%)	EMC MDF (%)
1	23 °C and 77%	15.0	14.0	23 °C and 65%	12.3	11.5
2	26 °C and 57%	11.1	10.2	26 °C and 57%	11.1	10.2
3	27 °C and 20%	5.8	4.9	27 °C and 20%	5.8	4.9
4	20 °C and 31%	7.5	6.6	20 °C and 31%	7.5	6.6
5	19 °C and 87%	18.5	17.5	19 °C and 65%	12.5	11.7
6	23 °C and 63%	11.9	11.0	23 °C and 63%	11.9	11.0

**Table 2:** Methods used in the literature to determine the moisture distribution M and/or diffusion D for wood particleboards.

Year	Author (s)	Expression	Advantage	Limits
1949	Crank and Park [25]	D = (0.049 a_2_)/t_0.5_	Easy in application and calculus.	Significant error. Many missed parameters.
1956	Crank [24]	D = (0.1976 l2)/t_0.5_	Easy in application and calculus.	Significant error. Many missed parameters.
1966	Luikov [26]	$\frac{M_{c}-M_{s}}{M_{0}-M_{e}}=\frac{1}{2}{Ki}_{m}\left(1+\varepsilon P_{n}K_{0}L_{u}\right)$ $\frac{T_{s}-T_{c}}{T_{a}-T_{0}}=\frac{1}{2}{Ki}_{m}\varepsilon K_{0}L_{u}$ *Kim*, *ε*, *P_n_*, *K*_0_ and *L_u_*: Variable parameters	Analytic resolution. General formula considering both mass and heat transfer.	Depending on a lot of parameters. Difference from specimen surface to center.
1974	Simpson [24]	$\frac{\partial S}{\partial T}=D\frac{\partial^{2}S}{\partial X^{2}}$ $\frac{{\overset{`}{S}}_{m}-S_{m}}{∆T}=D\left(\frac{S_{m+1}-2S_{m}+S_{m-1}}{{\left(∆X\right)}^{2}}\right)$ *S*: Averages of dealing with the concentration depending on the diffusion coefficient. ${\overset{`}{S}}_{m}$ and $S_{m}$ are the values of *S* at the points *X = m*(Δ*X*) at *T* = (*n* + 1) Δ*T* and *T* = *n* (Δ*T*), respectively, and *Sm* + 1 and *Sm* − 1 are the values of *S* at *X* = (*m* + 1)(Δ*X*) and *X* = *X* = (*m* − 1)(Δ*X)* at *T = n* (Δ*T*).	Series of approximations that are repeated until certain experimental and calculated values agree. Good match with experience results.	Large lumber of successive approximations. A high-speed computer is necessary to apply the method.
1977	Whitaker [31]	$\left(\frac{\partial S}{\partial t}\right)=\nabla .(D\nabla S)$ *D*: drying diffusion coefficient *S*: fractional moisture saturation $D=K_{\varepsilon }+\left[\frac{\partial \xi }{\partial S}\right]\frac{D_{eff}^{\left(1\right)}}{\varepsilon_{\gamma }}$	Analytic resolution. Based on simultaneous heat, mass, and momentum transfer in porous media.	Many assumptions (neglected parameters) and approximations to solve the problem. A lot of calculations.
1984	Siau [22]	$\overset{`}{D}=\frac{E^{2}L^{2}}{5.10t}$ *L* = thickness in direction of flow, cm; t = time, s. *E* = dimensionless term. *D’* = diffusion coefficient which includes diffusion coefficient *D* and surface emission coefficient *S*	Analytic equation. Simple to calculate.	Includes unknown variable S (surface emission coefficient).
1986	Stanish [32]	$\frac{\partial }{\partial t}\left(\rho_{a}\right)=-\frac{\partial }{\partial z}\left(n_{a}\right)$ (The conservation equation for air) ddtρaρmT=AMC	Simulated and experimental results revealed minor changes in the model parameter set.	Computational resolution by finite elements.
1989	Liu [29]	D=−0.165(a2)20.701dt0.5dE+2.05t0.5 S=0.701 D a2Dt/a22−0.196 $\frac{t_{0.5}}{0.2}\left(\frac{L}{2}\right)=\frac{\left(L/2\right)}{D}+\frac{3.5}{S}$	Use of surface emission coefficient *S* with diffusion *D* to obtain global phenomena understanding.	Significant error. Many missed parameters.
1991	Simpson et Liu [30]	D = A exp (B m) A and B are coefficients determined by non-linear regression	Treats high-level moisture. Easy to apply.	Proceeding by two approximations. A and B numerical value changing with approximation. Adjusted parameters by experience.
1993	Cloutier and Fortin [24] (based on [25,30,31] works)	${\left[K_{x}\right]}_{xi,tj}=\frac{{\left[\frac{\partial I}{\partial t}\right]}_{xi,tj}}{{\left[\frac{\partial \psi }{\partial x}\right]}_{xi,tj}}$ $I=\int_{xq=0}^{x}Cdx$ [*Kx*]*xi*,*tj* = effective water conductivity in the direction of flow x at position *xi* and time *tj*; [*∂I/∂t*]*xi*,*tj* = −(flow through plane *xi* at time *tj*); [*∂ψ/∂x*]*xi*,*tj* = *ψ* gradient at *xi* and *tj*. *C*: moisture concentration *ψ*: water potential (See Figure 1)	Combining analytic and experimental resolution. Less error and more precision than another formula.	Many notions and equations are to be considered. Needs a small gap between points, so more computing resources.
1992	L. Cai and S. Deku [35]	D’ = b0 + b1M + b2M2 + b3M3	The diffusion D’ was expressed as a function of water content M with a third-degree polynomial. Use of the FEM.	Neglects the resistance of the surface in the unsteady regime. Error about 10% between experiment and FEM.
1994	L. Cai, F. and Wang [37]	(1) The diffusion coefficients of moisture movement perpendicular (d) and parallel (d) (2) Usins Liu [29] and Siau [22] equations to determine the diffusion coefficient (3) Regression equation Y = b + aX a,b depending on parameters on materials and direction (4) Determinate a simple relationship between moisture M, Diffusion D, and time t, for each case (example: M = 1.4774 t 0.178 with correlation coefficient R = 0.9710)	Treat both steady-state and unsteady-state. Determines diffusion coefficient D for both parallel and perpendicular orientations. Determines the linear relationship between diffusion *D* and moisture *M* for each case.	No general equation (equation for each case). A lot of approximation. Uses experience and graphic analysis to determine diffusion d for the steady state (giving the results by direct values).
1996	Wu and Suchsland [13]	Determinate the moisture content MC in two ways and compare between them: Predicting: Equation (5) and *D* $D=\left(\frac{\pi L^{2}}{4}\right){\left[\frac{E_{2}{-E}_{1}}{\sqrt{t_{2}}-\sqrt{t_{1}}}\right]}^{2}$ $E_{i}={(W}_{i}-W_{1})/(W_{E}-W_{1})$ *W_i_* = specimen weight at time ‘‘*i*” *W*_1_ = initial specimen weight, *W_E_* = specimen weight at equilibrium, *L* = half specimen thickness. Measuring: Using Simpson’s [27] equation to determine the diffusion coefficient Comparing the two approaches	Good agreement between predicted and measured MC for three different panels. Demonstrates the backer can reduce the extent of the moisture differential and swelling stress imbalance.	The moisture profile is supposed uniform across the specimen thickness. Using experimental results that cannot fully create the assumed conditions.
2001	Wu Q, and Xiong M [36]	K (p,x) = 0.00431 − 0.00107p + 0.00192x + 0.0005p^2^ − 0.0192x^2^ + 0.000206.x.p	No big calculation and complex formula. Dedicated to MDF and particle boards.	A small range of temperature and humidity.

**Table 3:** Simulation parameters.

Parameter	Type	Definition	Value/Expression	Reference
T	Boundary conditions	Temperature (°K)	292.15–300.15	Table 1
RH	Boundary conditions	Relative humidity	20–65%	Table 1
T_ini	Initial conditions	Initial temperature (°K)	291.15	Present work
MC_ini	Initial conditions	Initial moisture content	18%	Present work
MC_fin	Boundary conditions	Final moisture content	EMC	Present work
D		Diffusion (m^2^/s)	Equation (15)	Present work
CP_Melamine		Specific heat capacity of melamine (J/kg·K)	6·714×T−604·53	[40,41]
CP_MDF		Specific heat capacity of MDF (J/kg·K)	3.867×T+103·1	[42,43]

**Table 4:** Mesh sensitivity study.

Test #	1		2		3		4		5		6		7		8		9	
	Min	Max	Min	Max	Min	Max	Min	Max	Min	Max	Min	Max	Min	Max	Min	Max	Min	Max
Element size (mm)	14	100	10.8	60	8	38	5.6	30	3.6	20	2	16	0.8	11	0.3	7	0.04	4
Calculation time	14 min 21 s		4 min 58 s		6 min 20 s		4 min 57 s		7 min 53 s		11 min 22 s		45 min 31 s		1 h 50 min 40 s		2 h 15 min 21 s	
Relative error	21.67%		19.22%		16.90%		15.28%		11.69%		4.70%		2.50%		0.6%		0.5%	

**Table 5:** Anhydride samples (MC = 0%).

Material	Dimensions (L × W × T) [mm]	Sample Numbers	Density (kg/m^3^)
**Melamine**	200 × 100 × 15.8	1P1, 1P2, 1P3, 1P4, 1P5, 1P6, 2P1, 2P2, 2P3, 2P4, 2P5, 2P6	728 (calculated)
**MDF**	200 × 200 × 12.7	1M1, 1M2, 1M3, 1M4, 1M5, 1M6, 2M1, 2M2, 2M3, 2M4, 2M5, 2M6	728 (calculated)

**Table 6:** Anhydride samples (MC = 0%).

MDF				Melamine			
First Test		Second Test		First Test		Second Test	
Sample	Weight (g)	Sample	Weight (g)	Sample	Weight (g)	Sample	Weight (g)
1M1	355.18	2M1	370.91	1P1	222.45	2P1	227.16
1M2	362.58	2M2	371.37	1P2	237.39	2P2	233.99
1M3	359.39	2M3	378.33	1P3	228.89	2P3	217.08
1M4	369.35	2M4	371.08	1P4	239.87	2P4	235.74
1M5	372.93	2M5	375.71	1P5	222.88	2P5	235.27
1M6	378.50	2M6	385.99	1P6	236.58	2P6	224.70

**Table 7:** Values of MC for melamine and MDF.

Time (Days)	MC (%) Melamine	MC (%) MDF
0.00	0.0%	0.0%
1.00	5.3%	6.6%
4.00	9.0%	14.8%
5.00	10.2%	15.4%
7.00	11.4%	16.1%
12.00	14.9%	17.3%
18.00	16.9%	17.9%
19.00	17.5%	18.1%
20.00	17.7%	18.1%
21.00	17.9%	18.2%

**Table 8:** Time required to reach the appropriate MC for each test condition.

Test #	Test Conditions	Melamine (Days)		MDF (Days)	
		12%	8%	12%	8%
1	23 °C and 65%	30	66	25	58
2	26 °C and 57%	13	33	7	19
3	27 °C and 20%	10.4	14.3	7.3	10.4
4	20 °C and 31%	5.4	12.6	7.4	16
5	19 °C and 65%	21	70	18	60
6	23 °C and 63%	23	68	18	54

**Table 9:** Numerical results of ANN network for melamine and MDF.

Material	ANN Architecture	MSE Training	MSE Validation	R	Linear Regression Model
Melamine	[3-10-7-1]	0.17	0.13	0.99956	Output = 1*Target + 0.011
MDF	[3-10-10-1]	0.08	0.33	0.99924	Output = 1*Target + 0.071

**Table 10:** Results of comparison between experimental results and simulation.

	Condition	1	2	3	4	5	6
Melamine	Minimum error	0.3%	1.7%	0.5%	2.7%	0.2%	0.9%
	Maximum error	18.6%	14.6%	20.7%	25.4%	20.8%	22.2%
	Average errors	9.6%	8.9%	10.8%	10.1%	13.1%	14.2%
MDF	Minimum error	1.1%	1.2%	0.6%	0.2%	0.9%	0.8%
	Maximum error	15.7%	15.5%	24.6%	10.3%	19.8%	22.2%
	Average errors	8.3%	10.7%	10.7%	4.4%	13.5%	14.2%

**Table 11:** Results of comparison between experimental results and ANN.

Condition	1	2	3	4	5	6
Melamine (8%) Experience	66	33	14.3	12.6	70	68
Melamine (8%) ANN	66.3	33.51	14.26	12.76	67.66	69.04
PE	0.46%	1.55%	0.27%	1.27%	3.35%	1.53%
Average	1.40%					
Melamine (12%) Experience	30	13	10.4	5.4	21	23
Melamine (12%) ANN	29.05	12.69	9.91	5.42	20.68	21.86
PE	3.17%	2.39%	4.70%	0.37%	1.52%	4.94%
Average	2.85%					
MDF (8%) Experience	58	19	10.4	16	60	54
MDF (8%) ANN	58.44	19.5	10.6	16.15	61.24	54.11
PE	0.77%	2.64%	1.88%	0.94%	2.07%	0.21%
Average	1.42%					
MDF (12%) Experience	25	7	7.3	7.4	18	18
MDF (12%) ANN	24.79	7.18	7.51	7.65	17.58	17.73
PE	0.85%	2.60%	2.81%	3.43%	2.31%	1.48%
Average	2.25%					

## Data Availability

Data is available from corresponding authors upon request.
